# DNA Aptamers as Molecular Probes for Colorectal Cancer Study

**DOI:** 10.1371/journal.pone.0014269

**Published:** 2010-12-10

**Authors:** Kwame Sefah, Ling Meng, Dalia Lopez-Colon, Elizabeth Jimenez, Chen Liu, Weihong Tan

**Affiliations:** 1 Department of Chemistry, Department of Physiology and Functional Genomics, Shands Cancer Center, UF Genetics Institute and McKnight Brain Institute, University of Florida, Gainesville, Florida, United States of America; 2 Department of Pathology, Immunology and Lab Medicine, University of Florida, Gainesville, Florida, United States of America; 3 H. Lee Moffitt Cancer Center & Research Institute, Tampa, Florida, United States of America; Aristotle University of Thessaloniki, Greece

## Abstract

**Background:**

Understanding the molecular features of specific tumors can increase our knowledge about the mechanism(s) underlying disease development and progression. This is particularly significant for colorectal cancer, which is a heterogeneous complex of diseases developed in a sequential manner through a multistep carcinogenic process. As such, it is likely that tumors with similar characteristics might originate in the same manner and have a similar molecular behavior. Therefore, specific mapping of the molecular features can be potentially useful for both tumor classification and the development of appropriate therapeutic regimens. However, this can only be accomplished by developing high-affinity molecular probes with the ability to recognize specific markers associated with different tumors. Aptamers can most easily meet this challenge based on their target diversity, flexible manipulation and ease of development.

**Methodology and Results:**

Using a method known as cell-based Systematic Evolution of Ligands by Exponential enrichment (cell-SELEX) and colorectal cancer cultured cell lines DLD-1 and HCT 116, we selected a panel of target-specific aptamers. Binding studies by flow cytometry and confocal microscopy showed that these aptamers have high affinity and selectivity. Our data further show that these aptamers neither recognize normal colon cells (cultured and fresh), nor do they recognize most other cancer cell lines tested.

**Conclusion/Significance:**

The selected aptamers can identify specific biomarkers associated with colorectal cancers. We believe that these probes could be further developed for early disease detection, as well as prognostic markers, of colorectal cancers.

## Introduction

Colorectal cancer (CRC) is the third most common cancer (10–15% of all cancers) and one of the leading causes of cancer-related deaths worldwide, with an estimated half a million deaths worldwide and over fifty thousand deaths in the United States alone.

CRC is a heterogeneous complex of diseases caused by destructive genetic/epigenetic alterations that accumulate in a sequential manner through a multistep carcinogenic process [1]. It is therefore likely that tumors with similar characteristics might originate in the same manner and have a similar molecular behavior. Since the molecular features of a given tumor reflect the mechanism(s) underlying disease development and progression, the implication for tumor classification is significant. For instance, molecular classification of leukemia and lymphomas has tremendously enhanced our understanding of these diseases [2], [3], [4]. The investigation of the molecular bases of two major syndromes, familial adenomatous polyposis (FAP) and hereditary nonpolypsis CRC (HNPCC), has led to the identification of two main pathways by which these molecular events can lead to CRC [5]. About 85% of CRCs arise from events that result in chromosomal instability (CIN), with aneuploidy and early inactivation of adenomatosis polyposis coli (APC). A further 15% result from processes that generate microsatellite instability (MSI), replication error or loss in the caretaker mismatch repair (MMR) function associated with HNPCC [6], [7], [8], [9]. Although we have improved our understanding of the molecular events underlying the development of CRC, no significant impact on patient care has resulted. Even though considerable progress that been made in the treatment of patients with CRC using folic acid (FA)-modulated 5-flurouracil (5-FU), about 50% of CRC patients eventually develop metastatic CRC (mCRC). However, the use of new chemotherapy agents, such as oxaliplatin and irinotecan, either alone or in combination with approved biological agents, such bevacizumab and cetuximab, promises to prolong survival [10], [11].

Therefore, in order to maximize the available treatments, it is critically important to gain even more insight into the molecular mechanisms underlying disease development and progression, as well as significantly improve our efforts to elucidate new therapeutically relevant targets and molecular markers. Such efforts will help expand and diversify disease management options. Studies have also shown that shifting disease detection to an earlier stage through mass screening and intervention at this stage can reduce the risk of death from CRC [12], [13]. These findings strongly demonstrate the clinical need for biomarkers for the early detection of CRC so that the disease can be effectively managed.

Genomic techniques, such as DNA microarray analysis, and proteomic methods, such as two-dimensional (2-D) electrophoresis and mass spectrometry, are now commonly used to elucidate the expression profiles of genes and proteins in cells, tissues and bodily fluids [14], [15]. Indeed, the identification of genes and proteins that are characteristically produced during the development of cancer can potentially uncover useful biomarkers that will aid in the management of CRC. Although proteomics have played a dominant role in the field of biomarker development [16] and will continue to do so, the current proteomic strategies have not generated enough markers for CRC.

Interestingly, CRC is one of the first cancers for which tumor markers were used to aid in disease management. For example, carcinoembryonic antigen (CEA) has been used extensively to determine prognosis and monitor both disease progression and therapy after curative resection. A high level of CEA in the serum is associated with cancer progression. However, even in the absence of cancer, high levels of CEA have been reported in conditions such as hepatitis, pancreatitis, inflammatory bowel disease and obstructive pulmonary disease. In addition, other cancers, such as pancreatic, gastric, lung and breast, have elevated levels of CEA, indicating the lack of specificity of this marker. Other markers, such as carbohydrate antigen 19-9 (CA19.9), CA242, metalloproteinases-1 (TIMP-1), Thymidylate synthase, p53, and *APC* gene, all lack the necessary sensitivity and specificity. To be clinically useful, a biomarker must be effective and have high predictive value [7], yet no such single biomarker exists. Although no consensus has been reached, it seems that the development of multiple biomarkers for detection and risk assessment of a single cancer is favored over single comprehensive biomarkers that can successively predict risk for any type of cancer [7]. This is even more important as multiplexing methods are becoming more of a norm than the exception.

Biomarkers that are directly associated with tumor cells as they are transformed from normalcy into malignancy will be of significant interest as these markers can be useful tools for mapping the molecular features of the diseased cells. The ability of probes to identify an important clinical specimen, such as exfoliated malignant colonocytes, rather than normal colonocytes, would be particularly important for CRC [16]. Specifically, once exfoliated cells from colonic mucosa have been sloughed into the stool, it has already been demonstrated that DNA from the stool can be isolated and subjected to a multi-target DNA analysis. This assay can currently assess 15 mutational hot spots, including k-ras, p53, APC, BAT-26 and L-DNA. Although DNA fecal markers are quite promising, they are not widely used in clinical settings and therefore probes that can specifically detect the cells will be important.

In the development of sensitive, selective molecular markers for CRC, cell-based aptamer selection holds significant promise by its potential to identify multiple useful markers in a relatively short time. During the last two decades, appreciable efforts have been made to develop markers for various types of cancers using a process known as the Sytematic Evolution of Ligands by Exponential enrichment (SELEX) [17], [18]. Through this process, numerous oligonucleotide probes (aptamers) have been generated that can bind specifically to proteins associated with membranes of different tumor cells [19], [20], [21], [22], [23], [24], [25], [26]. Aptamers are short, single-stranded oligonucleotides (DNA or RNA), typically <100 mer, that have the ability to bind to other molecules with high affinity and specificity. They have been generated against a variety of targets from small molecules [27], [28], [29], [30] and peptides/proteins [31], [32], [33], [34], [35], [36] to whole cells [19], [20], [21], [22], [23], [24], [25], [26] as well as bacteria, viruses and virus associated proteins [37], [38], [39], [40], [41], [42], [43]. The cell-based selection strategy generates aptamers that bind to unknown targets in their native state. Nevertheless, the target can still be identified through affinity extraction and mass spectrometry [44], [45]. To create more sensitive and selective probes for CRC, we have developed a panel of DNA aptamers that specifically recognize colorectal cancer cells. These probes were generated by cell-SELEX using colorectal cancer cultured cell lines DLD-1 and HCT 116. Initial binding studies by flow cytometry and confocal microscopy using these cultured cell lines show that most of our aptamers have high affinity and selectivity. Our data further show that these aptamers neither recognize normal colon cells (cultured), nor do they recognize a majority of the other cancer cell lines tested. Our findings clearly show that the probes identify specific membrane proteins associated with colorectal cancers. We believe that these probes could be further developed for early disease detection, as well as prognostic markers, of colorectal cancers.

## Results

**Figure 1:**
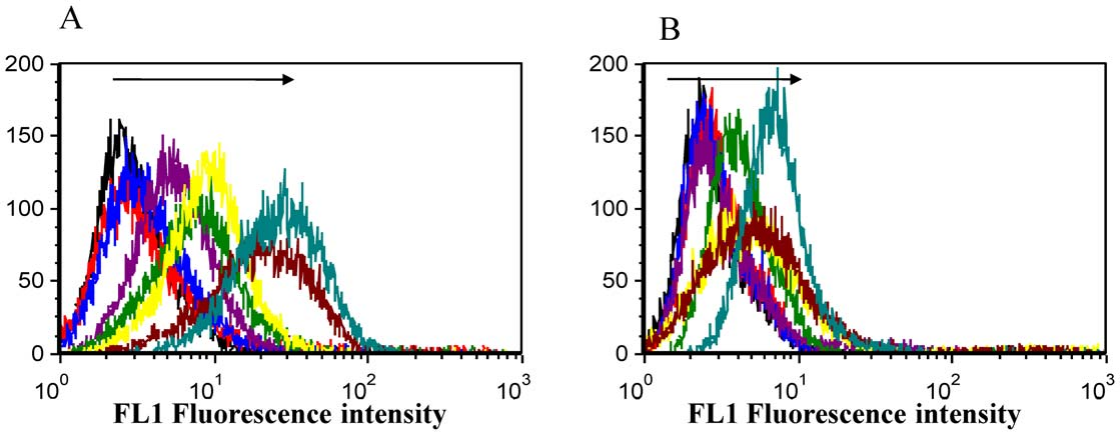
Enrichment of selected DNA pools of target DLD-1 (A) and control HCT 116 (B) during selection and monitored by Flow cytometry. Direction of arrows shows increasing rounds of selection from the 5^th^ -16^th^ pool. As selection progressed there was higher corresponding increase in fluorescence intensity of the target than the control. The sudden increase in fluorescence signal of the control from 14^th^ to 16^th^ round without corresponding increase in the target indicates higher enrichment.

Over the last decade, several aptamers have been developed for different targets, including purified molecules, as well as complex targets, such as the live cells of different cancers. We have used the cell-based SELEX strategy to generate a panel of DNA aptamers for colorectal cancers. A random ssDNA pool (approximately 10^14^) was subjected to sequential binding and elution to select from the pool DNA sequences having the ability to bind to surface markers of the target cell. DLD-1 and HCT 116 were used as targets with HCT1116 and HT-29 as respective controls in separate selections. The introduction of counter selection provided the opportunity to eliminate, to the extent possible, common surface markers, while at the same time enriching differential markers on the target cells. The DNA pool collected after each round of selection was amplified by PCR, and the product was used to prepare ssDNA for the next round of selection. In this selection strategy, the incubation of the DNA pool with the cells was performed in culture dishes (cell monolayers). The enrichment of the selection pool through successive selection was monitored by flow cytometry. In order to use the cells for flow cytometry, cells were cultured overnight and dissociated using short time (30 sec–1 min) trypsin treatment and/or non-enzymatic cell dissociation solution (MP Biomedicals). Short time treatment with trypsin did not have any observable effect with respect to the ability of the selection pool to recognize the cells, since no difference between the signal intensities of the trypsin and the non-enzymatic treatment options was observed (Text S1, figure S1). Peak shift (increase in the fluorescence intensity as compared to the library) is an indication of fluorescence intensity of the cell as a result of the labeled DNA sequence binding to the cells (Figure 1). With the increasing number of selection cycles, there was a steady increase in the fluorescence intensity of the target cells, indicating that DNA sequences with better binding to the target cells were being enriched. However, there was no significant peak shift with the control cells, especially in the early and mid rounds of selection. By the 14^th^ round of selection, there was a significant increase in fluorescence signal of the target as compared to the control cells. An additional two rounds (16^th^ round) led to an increase in the signal enhancement of the control, but no significant increase for the target. This is possible at the terminal end when selection is continued further even when there is no significant signal difference between the successive pools to the target. The highly enriched pools were cloned and the positive clones sequenced. Sequence analysis provided potential DNA aptamer candidates grouped into families based on their sequence homology. About 8 distinct homologous families and many randomized sequences were identified for the DLD-1 selection. The number of sequences in the distinct homologous families ranged from 6–110 (Table 1) with few base mutations within a family. Representative sequences from the different families were chosen to test their interactions with the target.

**Table 1:** Representation of different homologous families after cloning and sequencing and alignment of DLD-1 selection.

Family	Number of sequences	Percentage of total sequences
1	110	25.5
2	46	10.7
3	22	5.1
4	30	7.0
5	23	5.3
6	26	6.0
7	6	1.4
8	9	2.1
9	26	6.0
Random	<6	30.9

Members in each family differ in few numbers of bases.

**Figure 2:**
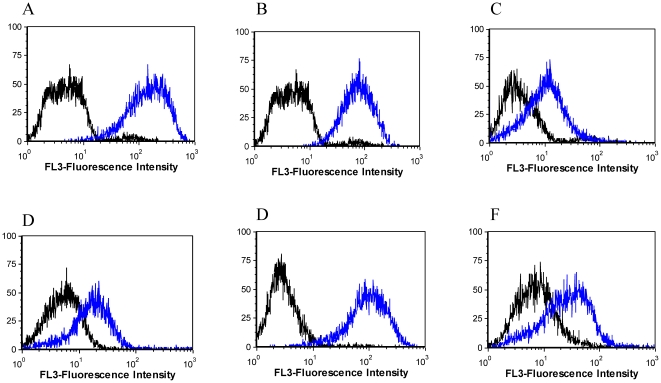
Flow cytometry histograms showing the binding of representative aptamer candidates screened against the target DLD-1 cells. Cells were dissociated with non-enzymatic dissociation solution. The cells were washed and incubated with different aptamer candidates (blue histogram). The fluorescence signal was detected by streptavidin-PE-cy5.5. The unselected library was used as background fluorescence signal (black histogram). All the aptamers A (KDED2), B (KDED5), C (KDED7), D (KDED15), E (KDED19), F (KDED20).

The rolling cycle amplification (RCA) products from sequencing were used for the initial screening. The products from the chosen wells were diluted with 100 µl H_2_0 and used for PCR amplification. The PCR products were used to prepare ssDNA and then used for binding assays. Two sequences having at least one base mutation were chosen from families with sequences greater than 20 (Table 1). The use of the RCA product provided us with a faster method of screening for potential aptamer candidates. The sequences with binding signal greater than that of the control (Text S1, figure S2) were chemically synthesized and labeled with fluorescein isothiocynate (FITC) or biotin at the 3′ end. All synthesized sequences were purified by HPLC and then quantified. The binding assays were performed using flow cytometry, as described, and the binding signal was directly detected in FL1 for FITC probes, FL2 for streptavidin-conjugated PE or FL3 for streptavidin-conjugated PE-Cy5.5. The initial binding assays with the synthesized sequences named KDED1 to KDED20 revealed many sequences binding to the cell target (Figure 2). Some of the sequences belonging to the same family, and therefore envisaged to be binding to the same target, showed different binding signal strengths to the target. These include KDED2/KDED15; KDED1/KDED5; KDED9/KDED10, KDED3/KDED19 and KDED7/KDED18.

**Figure 3:**
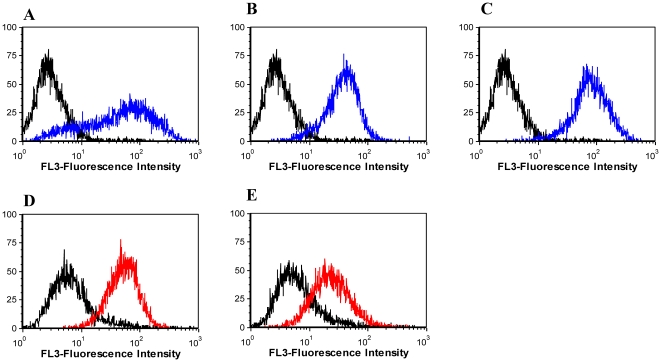
Screening of selected aptamers candidates in the alternate selection against DLD-1 (Blue) and HCT 116 (red) using flow cytometry. A (KC2D3), B (KC2D4), C (KC2D8). D (KCHA10), E (KCHB10).

The co-current selections, using DLD-1 (without negative selection) and HCT 116 as targets, also generated the following aptamers: KC2D3, KC2D4, and KC2D8 for DLD-1: KCHA10 and KCHB10 for HCT 116 (Figure 3).

**Figure 4:**
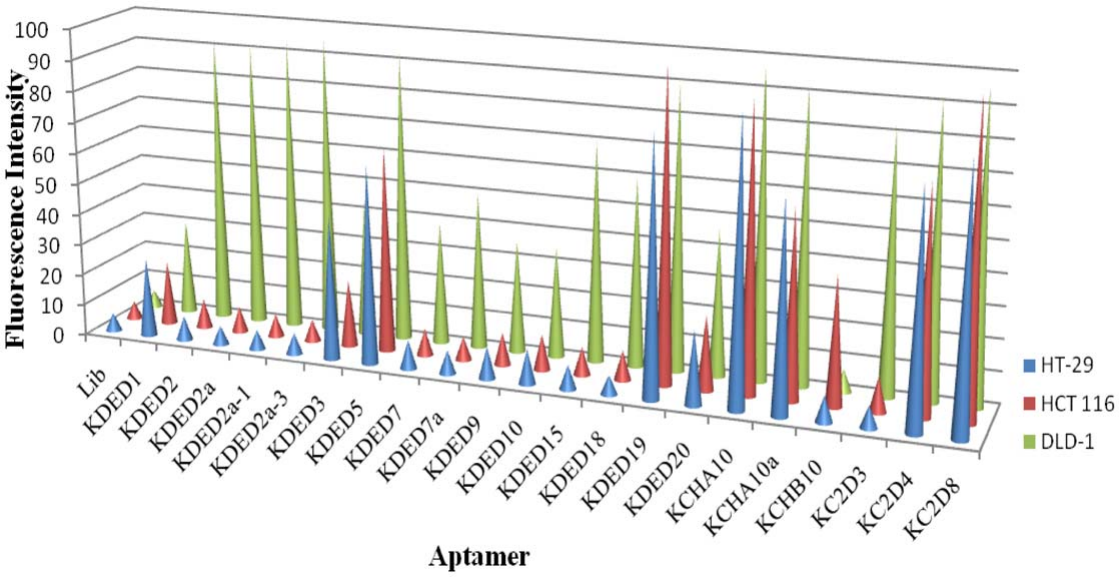
Pictorial representation of the recognition pattern of the different aptamers generated against DLD-1 and HCT 116. Binding assays were performed with DLD-1, HCT 116 and HT-29. The background fluorescence intensity of the library was set below 5% and the fluorescence signal of the individual aptamers was then determined and used in pictorial representation as shown.

All the developed aptamers were further tested with all the colorectal cancer cell lines used in this study. The following aptamers showed recognition only to the cell line used for the selection: KDED2/KDED15, KDED7/KDED18, KDED9/KDED10 and KC2D3 (DLD-1) and KCHB10 (HCT 116). Figure 4 shows the pictorial representation of the interaction between the individual aptamers and the three different colorectal cancer cell lines. The height of the cone represents the percentage of cells that had fluorescence intensity above the control library with the threshold set at 5% fluorescence signal intensity.

**Figure 5:**
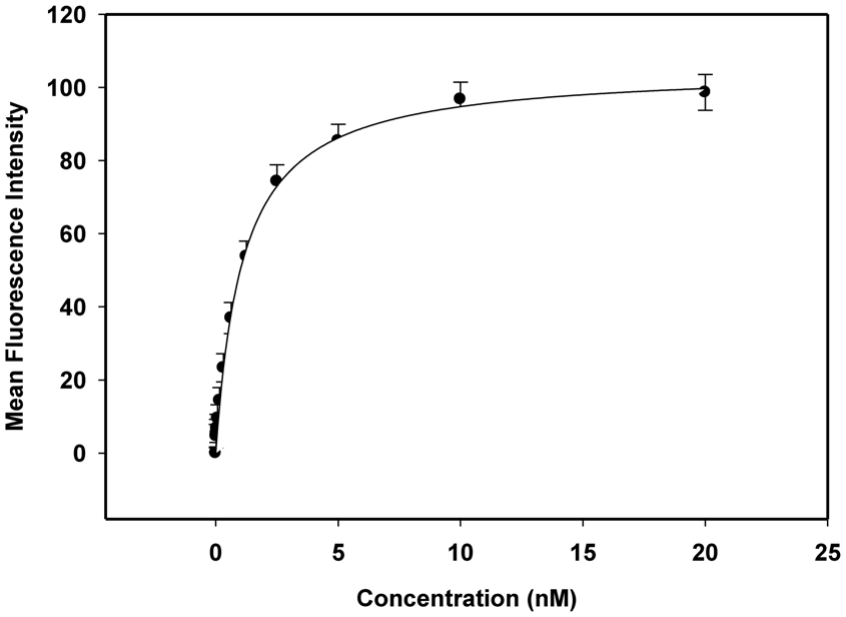
Binding curve of KC2D8 aptamer with DLD-1 cells. Cells were incubated with varying concentrations of Biotin-labeled aptamer in duplicate. The florescence signal was detected with streptavidin-PE-cy5.5. The mean fluorescence intensity of the unselected library (background binding) at each concentration was subtracted from the mean fluorescence intensity of the corresponding aptamer. The actual fluorescence intensity was fitted into Sigmaplot to determine the apparent Kd.

We then determined the apparent dissociation constants (Kd) for the selected aptamers, which ranged between 0.68 nM and 302 nM (Table 2 and Figure 5). In order to enhance synthesis efficiency and also increase the flexibility of chemical manipulation, some of the aptamers were truncated, and the binding strength and apparent Kds of the truncated sequences were also assessed. Before each truncation, the possible structures of each aptamer sequence were predicted using Integrated DNA Technologies oligoanalyzer under the selection conditions. The most favorable hairpin structures were selected and the hang over bases at the 3′ and/or the 5′ end removed, each at a time. Series of truncations were made and each of these was finally tested with the positive cells to assess the binding. The truncation of KCHA10 (KCHA10a) did not change the properties of the aptamer. However, significant improvement of binding affinity was observed with KDED7 truncation (KDED7a) and the various forms of KDED2 (KDED2a, KDED2a-1 and KDED2a-3), as demonstrated in the improved affinities of the truncated versions (Table 2). For instance, the Kd of KDED7 improved from 157.3 nM to 46.8 nM, about 3-fold improvement, while KDED2 improved from 191.9 nM to 29.9 nM, a 6-fold improvement. The rest did not show observable binding to the target. In almost all the assays reported, we have used all the individual sequences (truncated and full- length) of KDED2, since the difference in binding affinity can influence the sensitivity of a particular assay.

**Table 2:** Different aptamer sequences and their corresponding apparent dissociation constants (Kd).

Aptamer	Sequence	Kd (nM)
KDED1	AGAGACCCTGACTGCGAACTAAACAAAATACGAGCAGGGAGACTTCTATCCGATTGTTGGACACGGTGGCTTCTT	ND
KDED2	AGAGACCCTGACTGCGAAAACTGCTATTACGTGTGAGAGGAAAGATCACGCGGGTTCGTGGACACGGTGGCTT	191.9±35.2
KDED2a		139.2±25.0
KDED2a-1		50.5±11.4
KDED2a-3		29.2±6.4
KDED3	AGAGACCCTGACTGCGAAGGGGTGGTTTTCAAAGAGTCTTGCCTGACTCCCCTGTGGTGGACACGGTGGCTTCTT	125.7±6.0
KDED5		4.8±2.6
KDED7	AGAGACCCTGACTGCGAAGCGGACGCACTTTTAGCAAGCAAGTCGACAATGGAGGTTTTGGACACGGTGGCTTCTT	157.3±6.2
KDED7a		46.8±8.1
KDED9		ND
KDED10	AGAGACCCTGACTGCGAAGCAACTGATGCTAGAACTGTGTGGGGTTTGGGGTATAATTTGGACACGGTGGCTTCTT	ND
KDED15		302±25.0
KDED18	AGAGACCCTGACTGCGAAGCGGACGCACTTTTAGCAAGCAAGTTGACAATGGAGGTTTTGGACACGGTGGCTTCTT	ND
KDED19	AGAGACCCTGACTGCGAAGGGGTGGTTTTCAAAGAGTCTTGCCTGACTCCTCTGTGGTGGACACGGTGGCTTCTT	38.6±6.7
KDED20	AGAGACCCTGACTGCGAATAGGTTGGATAGGGATGGTAGAGCAGGCTAAGCACTTTTTTTTATTGGACACGGTGGCTTCTT	41.6±15.5
KCHA10		21.3±1.7
KCHA10a		28.2±2.7
KCHB10	ATCCAGAGTGACGCAGCAGATCTGTGTAGGATCGCAGTGTAGTTGACATTTGATACGACTGGCTGGACACGGTGGCTTAGT	3.9±0.4
KC2D3	ATCGTCCGCCACCACTACTCGGGAAAGGAACAAACTGCTATTAGGTCGCAGGCCGGTGAGACTGCCTGCCGATGT	32.1±3.4
KC2D4	ATCGTCCGCCACCACTACTCCCACTGGTAGCCATTCCGCCCTTAACCGGGCCATCGTGAGACTGCCTGCCGATGT	54.3±7.9
KC2D8		0.7±0.2

We further examined the selectivity by testing the interaction between aptamers and cultured normal cell lines, as well as cell lines from other cancers, including leukemia, lung, ovarian, brain and cervical cancer. As shown in Table 3, among the tested aptamers, KDED19 and KC2D8 showed significant signal intensity above the control library to some of the cell lines. For instance, there was significant recognition (≥ +++) of KDED19 to CAOV3, TOV21G, CEM and H661, whereas Hela and U87MG showed reduced signal (Table 3). KC2D8 showed a recognition trend similar to that of KDED19 with the cell lines tested (Table 3). There was no observable signal from the other aptamers with any of the cell lines tested, including the normal colon cell lines (CCD18Co and FHC) and fresh human colon cells (Text S1, figure S3). This implies that most of the developed aptamers are specific to colorectal cancers (Table 3). This observation is significant as it provides many possible options for further development of these aptamers for colorectal cancer studies. Because of the similar binding pattern observed with KDED19 and KC2D8, especially the signal with CEM, we decided to use Sgc8 against these aptamers in the subsequent competition assays to preliminary verify the target molecule.

**Table 3:** Recognition of aptamers with different cancer cell lines.

	KDED2	KDED5	KDED7	KDED10	KDED19	KDED20	KC2D3	KC2D8	KCHA10	KCHB10
**DLD-1**	+++++	+++++	++	++	+++++	++	++++	+++++	+++++	-
**HCT 116**	-	+++	-	-	+++++	+	-	+++++	+++++	++
**HT-29**	-	+++	-	-	++++	+	-	++++	+++++	-
**CCD18Co**	-	-	-	-	ND	ND	ND	ND	ND	ND
**FHC**	-	-	-	-	ND	ND	ND	ND	ND	ND
**HL60**	-	-	-	-	-	-	-	-	-	-
**NB4**	-	-	-	-	-	-	-	-	-	-
**K562**	-	-	-	-	-	-	-	-	-	-
**KG-1**	-	-	-	-	-	-	-	-	-	-
**CCRF-CEM**	-	-	-	-	++++	-	-	++++	-	-
**Ramos**	-	-	-	-	-	-	-	-	-	-
**Hela**	-	-	-	-	+	-	-	+	-	-
**NCI-H23**	-	-	-	-	ND	ND	ND	ND	ND	ND
**H1975**	-	-	-	-	ND	ND	ND	ND	ND	ND
**CAOV3**	-	-	-	-	+++	-	-	ND	-	-
**TOV21G**	-	-	-	-	++++	-	-	ND	-	-
**HBE 135**	-	-	-	-	-	-	-	ND	-	-
**H661**	-	-	-	-	++++	-	-	ND	-	-
**Ludlu**	-	-	-	-	-	-	-	-	-	-
**U87MG**		-		-	++	-	-	++	-	-

Selectivity study to assess the recognition of selected aptamers to different cell lines including colorectal cancer (DLD-1, HCT 116, HT-29), normal colon (CCD18Co, FHC), leukemia (HL60, NB4, K562, KG-1, CCRF-CEM, Ramos), lung cancer (NCI-H23, H1975, H661, Ludlu), cervical cancer (Hela), ovarian cancer (CAOV3, TOV21G), brain tumor (U87MG) and normal epithelial (HBE 135). A threshold of fluorescence intensity of PE-cy5.5 in the flow cytometry analysis was set such that the control library showed 5% of cells above the threshold. After binding the aptamer signal was evaluated by the percentage of cells above the threshold such that all signals below 10% were considered as background and designated ‘-’. The rest were assigned as follows; 11-30%, +; 31-50%, ++; 51-70%, +++; 71-85%, ++++ and >86, +++++. The final concentration of each aptamer was 250 nM.

In general, the cell-SELEX strategy produces different kinds of aptamers for different targets and/or the same target present on the extracellular surface of the cells. We therefore performed competition assays to assess if any of these aptamers, especially those from different families, could influence the binding of the other. Obviously, it was reasonable to assume that sequences synthesized from the same family will compete; also, sequences that bind different cells will not compete among themselves. For instance, it is possible for KDED2 to compete with KDED15, but it is unlikely that it will compete with KDED5 or KCHA10. On this basis, we performed competition of KDED2 (FITC) against KDED7, KDED10, KDED18, and KC2D3 (unlabeled), and KCHA10 (FITC) against KDED5, KDED20, KC2D4, KDED19 and KC2D8 (unlabeled), ending with KC2D4, KC2D8 and KDED19 against CEM aptamer Sgc8 (unlabeled). Ten-fold excess of the unlabeled competitor was first incubated with DLD-1 before the introduction of the FITC-labeled aptamer. This ensured competitive advantage and the potential to saturate and block the binding of the second aptamer if they both bound the same target or if the binding of one influenced the binding of the secondary aptamer. Unlabeled KDED2 and KCHA10 were used in these assays as positive control. KDED2 binding was not influenced by any of the aptamers tested against it. Similarly, we did not observe any competition between KCHA10 and any competing aptamers. However, there was significant influence on the binding of KDED19, KC2D4, and KC2D8 in the presence of 10-fold excess of unlabeled Sgc8 (Text S1, figure S4). This result suggests that these aptamers may be binding to the same target as Sgc8. This further suggests that KDED19, KC2D4 and KC2D8 will compete among themselves, although we did not perform such competition assay. These aptamers were developed using DLD-1, which did not have the initial blocking using Sgc8. This supports the idea that it is practicable to block a known marker in order to allow the development of probes for targets of interest.

**Figure 6:**
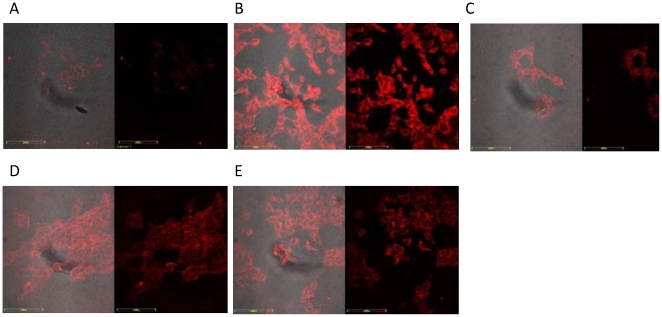
Confocal images of aptamers staining with cultured DLD-1 cells. Cells were incubated with aptamer conjugated with biotin and the binding event was observed with PE-conjugated streptavidin. A (unselected library showing the background binding); B (KDED2); C (KDED3); D (KDED5) and E (KDED7).

Immunohistological imaging and fluorescence microscopy have been widely used in the study of solid tumors and, in particular, colorectal cancers. Therefore, we also assessed if these aptamers could be used for tumor imaging with the positive cell line. In this preliminary study, we used cultured cell lines. Here we performed binding assays in culture dishes similar to the selection protocol, but with cell confluence of over 60%. After washing, the signal was detected with PE-streptavidin conjugate or streptavidin-Alexa Fluor 633. Figure 6 shows the confocal images of KDED2, KDED3, KDED5 and KDED7 detected with PE-streptavidin. There was significant signal strength of the tested aptamers compared with the unselected library. The signal pattern shows that the aptamers bound to the surface of the cells attached to the culture dish.

**Figure 7:**
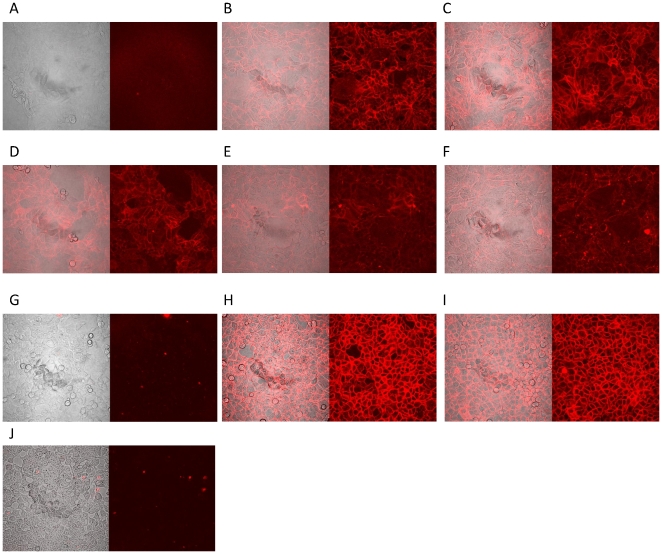
Confocal images of aptamers staining with cultured DLD-1 (A–F) and HCT 116 (G–J). Cells were incubated with aptamer conjugated with biotin and the binding event was observed with AlexaFluor 633 conjugated streptavidin. Unselected library shows the background binding. Aptamers show significant binding over the background signal. For DLD-1 cells, A =  unselected library; B  =  KCHA10; C  =  KDED19; D  =  KC2D8; E  =  KDED18; F  =  KDED20 and for HCT 116 cells, G  =  unselected library; H  =  KCHA10; I  =  KC2D8 and J  =  KDED2a-3.

Similarly, significant fluorescence signal was observed from streptavidin-Alexa Fluor 633 conjugates when DLD-1 was used against the other aptamers, including KCHA10, KC2D8, KDED18, KDED19, and KDED20 (Figure 7), as well as HCT 116 cells with KCHA10 and KC2D8. As expected, KDED2 did not bind HCT 116. The intensity of the fluorophore signal followed the same pattern as the flow cytometry. Interestingly, KC2D8 showed a stronger signal with HCT 116 than with DLD-1.

**Figure 8:**
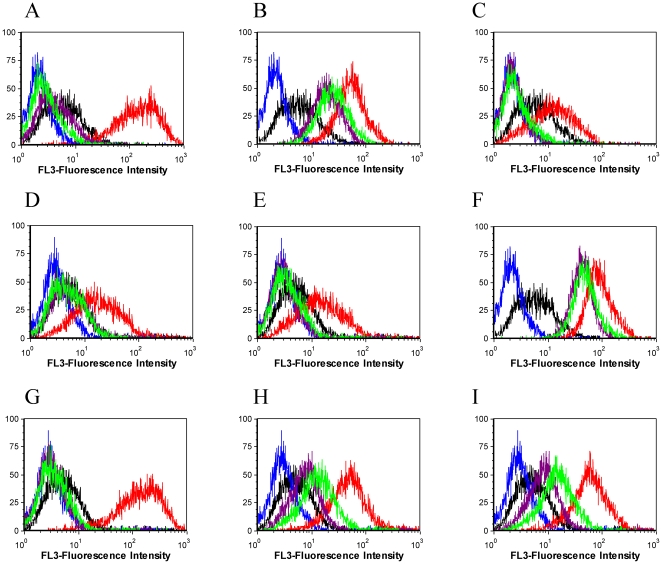
Preliminary determination of cell surface molecule that binds to aptamer. Cells were treated with trypsin and proteinase K for 15 min and then incubated with aptamer. The untreated cell incubated with aptamer was used as positive control. Black histogram, (unselected library with untreated cells); red (aptamer with untreated cells); blue (unselected library with trypsin treated cells); purple (aptamer with trypsin treated cells) and lime (aptamer with proteinase K treated). The following aptamers were assessed; A (KDED2); B (KDED5); C (KDED10); D (KDED18); E (KDED20); F (KCHA10); G (KC2D3); H (KC2D4), I (KC2D8). With the exception of KDED5 and KCHA10 which reduced only minimally, the signal of all others reduced significantly.

It is usual to assume that aptamers selected against tumor cell lines bind to surface proteins. This has been demonstrated in most of the SELEX protocols involving tumor cell lines [44], [45]. In order not to wholly translate any one SELEX data to the other, we performed assays to preliminarily determine the molecules on the cell surface to which the selected aptamers would bind. In this assay, the DLDL-1 cells were treated with trypsin and/or proteinase K for 15 min at 37°C. After the incubation period, the protease activity was stopped with the addition of ice cold culture medium containing FBS. The cells were quickly washed twice by centrifugation and then incubated with the aptamers. The untreated cells were used as positive control. Figure 8 shows the response of the aptamer binding after the protease activity. Except for KDED5 and KCHA10, all the other aptamers lost recognition in both trypsin- and proteinase K-treated cells. The fluorescence signals reduced to the background, indicating that the treatment of cells with the proteases caused digestion of the target protein. For KDED5, there was significant reduction in signal intensity, but the KCHA10 signal reduced only marginally, indicating that the targets were not wholly affected by the treatment.

We envisaged two possible causes: 1) insufficient time of protease treatment and/or 2) target molecules having significant portions other than protein, such as carbohydrate or heavily glycosylated protein not wholly exposed to protease digestion. In response, we performed long time (30 min and 1 hour) protease treatment with higher concentrations of proteinase K, as well as glycosidase treatment. The increase in protease concentration, as well as long incubation time, did not affect the binding of these two aptamers. In addition, the incubation of the cells with O-linked and N-linked glycosidases followed by protease treatment did not significantly influence the binding of these two aptamers. We believe that the glycosidase assays cannot be conclusive since we did not find literature to support the efficiency of this assay using cultured cell lines instead of pure glycoprotein.

The development of aptamers that will bind to the target at varying conditions, especially at physiological temperature and in culture medium, is important. This will increase the flexibility with which these aptamers can be adopted and implemented in many assay platforms. We therefore assessed the binding of the aptamers at 37°C, in culture medium, and under both conditions simultaneously. As shown in figure S5 (Text S1), KDED2, KDED2a, KDED2a-1 (same aptamer, but different sequence lengths) and KCHA10 maintained significant binding to DLD-1 cells. The signal strengths were not significantly different from the assays at 4°C. On the other hand, the other aptamers had reduced signal intensity to DLD-1. The signal difference may be a result of the differential affinity of individual aptamers. For instance, KDED2 (better affinity) and KDED15 belong to the same family, but KDED15 did not show significant binding at 37°C. In the RPMI-1640 experiments, KEDE2, KDED2a and KDED2a-1 showed equal binding strength, even when the incubation was performed at 37°C. KDED5 and KCHA10 showed reduced, but significant binding, as opposed to the remaining aptamers (Text S1, figure S6), some of which showed almost no binding. This observation is important in the further development of aptamer-based assays, such as targeted therapy, apoptotic and viability assays at physiological conditions.

## Discussion

Systematic evolution of nucleic acid probes for molecular recognition has generated a large number of useful aptamers for various applications in diverse fields, including biotechnology, biomedicine, pharmacology, microbiology and chemistry. We have used cell-SELEX to generate a panel of DNA aptamers that have specific recognition to colorectal cancers. In this report, we used DLD-1 and HCT 116 as the target cell lines, and the selection was performed in the culture dish. We believe that this system is an accurate representation of the native state of the surface markers. In one of the selections with DLD-1, we introduced negative selection with the HCT 116 cell line with the aim of selecting a panel of aptamers with diverse recognition patterns to colorectal cancers. Traditionally, in order to ensure the efficiency of negative selection, the control cell line is often in 5- to 10-fold excess of the target cell line. However, in these schemes, because the selection was done in the culture dish, the incubation volumes restricted the size of culture dish we could use. Consequently, only about 2-fold excess of control cell line was used. We therefore envisaged that the ratio of the positive and the negative cell lines was insufficient to potentially eliminate a significant number of the sequences binding to the common markers. However, we believe that it provided the opportunity for us to enrich for aptamers that could have differential binding patterns to colorectal cancer cell lines.

Sequential binding and elution of the DNA library pool with positive and negative cells eventually produced DNA-enriched pools that potentially bind to the target with high fluorescence signal intensity, but with minimal recognition to the control. In all flow cytometry binding assays for selected pools and aptamer candidates, cells were removed from culture dishes with either short time trypsin treatment (30 sec –1 min) at room temperature or by using commercial non-enzymatic dissociation buffer. We noticed that mild short time enzymatic treatment did not have any observable effect on the binding of any of the aptamers and that the fluorescence signal was similar to that of the cells in the non-enzymatic buffer. Consequently, we preferred using the trypsin in most of the assays since the non-enzymatic buffer was slightly harsh on cells, especially when left for a prolonged time. We generated a panel of DNA aptamers with diverse binding patterns to colorectal cancer cell lines. All four aptamer families (KDED2/KDED15; KDED7/KDED18; KDED9/KDED10 and KC2D3) showed specific binding to only DLD-1 cells, but not to the other colorectal cancer cell lines or the other cancer cell lines tested. In addition, one aptamer, KCHB10, showed specific binding to only HCT 116 cells. The other representative aptamer families, KDED3/KDED19; KDED1/KDED5, KDED20; KCHA10, KC2D4 and KC2D8, however, showed recognition to other colorectal cancer cell lines (HCT 116 and HT-29). CRC is a heterogeneous complex of diseases [1]; therefore, it is useful to find aptamers that have differential binding patterns. The importance of developing such panel of disease probes is that a combination of them can give high predictive value of disease management procedures [7], [46]. Such approach has been successfully implemented by using proteomic profiling to detect the risk of ovarian cancer [47]. A similar approach was demonstrated by detecting DNA from CRC stool sample using a multi-target DNA analysis panel. We believe that these sequences, when further developed, will contribute to efforts in developing more effective and reliable CRC disease management regimens.

Our flow cytometry data correlated with confocal microscopy imaging. We observed that aptamers which had high signal intensity by flow cytometry also produced brighter fluorescence signal by confocal microscopy. This is very important because of the fast turnaround time with flow cytometry coupled with its high sensitivity compared with immunohistochemical staining of tissue sections. This is supported by the study carried out by [48] which showed sensitivity of tissue-dissociated cells by flow cytometry compared to immunohistochemical staining. In their report, the mean fluorescence intensity of anti-EP4, anti-HLA-ABC, anti-HLA-DR and anti-CD80 was not affected by the enzymatic dissociation solution. However, although the mean fluorescence of anti-CD54 was reduced by 30% after 1 hour of enzymatic treatment, the signal was still significantly higher above the control. Therefore, in the future, we envisage that clinical CRC specimens can be dissociated and that the cell suspension can be used in flow cytometry analysis for faster and easier disease assessment or diagnosis.

The role of membrane and membrane-associated proteins in invasive and metastasis potential of tumor cells can be a major prognostic indicator in many cancers. Cell-SELEX can play a major role in identifying such potential markers through the development of aptamer probes. Preliminary determination of the target surface molecules of these aptamers indicates that they bind to membrane proteins. With the exception of KDED5 and KCHA10, which marginally reduced in signal strength, the rest of the aptamers completely lost recognition after protease treatment, meaning that the target was digested by the treatment. We envision that KDED5 and KCHA10 might be associated with glycoproteins which might be shielded by an extensive glycosylation, although the results from the glycosidase assay do not support it. We believe that this assay was not effective enough when adopted for whole live cells.

CRC is one of the first cancers to use tumor markers to aid management. Generally, however, because of the lack of very specific tumor markers, the needed advancements have not been realized. While the leading marker, CEA, has been used extensively to determine prognosis and to monitor disease progress and therapy after curative resection, it is not sufficiently specific based on its elevated level in other conditions, such as hepatitis, pancreatitis, inflammatory bowel disease and obstructive pulmonary disease, as well as pancreatic, gastric, lung and breast cancer. Similarly other markers such as KRAS had been suggested as prognostic markers, but KRAS mutations have been observed in several cancers and its importance as prognostic marker is still controversial. It is therefore important to observe that most of the aptamers developed in this report show significant specificity to CRC. Except for KDED19, KC2D4 and KC2D8, which bind to most of the cell lines tested, the rest of the aptamers are very specific to only CRC, with some recognizing a particular CRC cell line, as demonstrated by the specificity assays, but none of them bound to the normal fresh colon and normal colonic epithelial cell line, demonstrating that the target markers of these aptamers are disease-related. This initial characterization of the selected aptamers is very important since it provides key information regarding the potential use of these markers, though further studies may be necessary to determine their diagnostic and prognostic significance.

## Materials and Methods

### Cell lines and cell culture

Colorectal cancer cell lines DLD-1 (Dukes' type C colorectal adenocarcinoma), HCT 116 (colorectal carcinoma) and HT-29 (colorectal adenocarcinoma) were purchased from American Type Cell Culture (ATCC) and used for initial selection assays. Other cell lines used in this study to assess selectivity and the recognition pattern of the selected sequences include HL60 [26], K562 [26], NB4 [23], KG-1 (ATCC), Ramos [23], CCRF-CEM [23] (leukemia); Hela, cervical (ATCC); NCI-H23 [25], H1975 (ATCC) (lung) and FHC, CCD-18Co (ATCC) (normal colon). Cells were maintained in culture with RPMI-1640 containing 10% heat-inactivated FBS (Invitrogen) and 100 Units/mL penicillin-streptomycin (Cellgro) for DLD-1, HL60, NB4, K562, Ramos, CCRF-CEM, and Hela. Both HCT 116 and HT-29 cells were maintained in McCoy's 5A culture medium containing 10% heat-inactivated FBS and 100 Units/mL penicillin-streptomycin. FHC was maintained in 45% Ham's F12 medium; 45% Dulbecco's modified Eagle's medium, 25 mM HEPES; 10 ng/ml cholera toxin; 0.005 mg/ml insulin; 0.005 mg/ml transferrin; 100 ng/ml hydrocortisone; 10% FBS and 100 Units/mL penicillin-streptomycin. KG-1 cells were maintained in IMDM with 20% FBS and 100 Units/mL penicillin-streptomycin. All cultures were incubated at 37°C under a 5% CO_2_ atmosphere. Normal clinical colon tissue was obtained from the Department of Pathology, Shands Hospital, University of Florida.

### Oligonucleotides

Random DNA primers and libraries were designed using the Integrated DNA Technologies (IDT) software. The forward primer was labeled at the 5′ end with FITC, and the reverse primer was labeled with biotin at the 5′ end. Different primers and library sets were used for different selections to avoid cross contamination. All sequences, including aptamer sequences obtained after sequencing, were synthesized by standard phosphoramidite chemistry using a 3400 DNA synthesizer (Applied Biosystems) and purified by reverse phase HPLC (Varian Prostar). Before the selection process, the PCR amplification conditions of the primers and libraries were optimized. All PCR mixtures contained 50 mM KCl, 10 mM TrisHCl (pH 8.3), 1.5 mM MgCl_2_, dNTPs (each at 2.5 mM), 0.5 µM each primer, and Hot start *Taq* DNA polymerase (5 units/µl). Amplifications were carried out in a Biorad 1 Cycler at 95°C for 30 sec, 57.0°C for 30 sec, and 72°C for 30 sec, followed by the final extension for 3 min at 72°C. The FITC-coupled sequences were used to continue and monitor progress of selection by flow cytometry.

### Experimental procedure of cell-SELEX

In the first selection, DLD-1 was used as the target with HCT 116 as the control. Both cell lines grow as adherent monolayer. For the first round of selection, DLD-1 was cultured in a 100 mm ×20 mm culture dish to >95% confluence. Cells were washed in the dish with washing buffer (4.5 g/liter glucose, mM MgCl_2,_ dissolved in Dulbecco's PBS with magnesium chloride and calcium chloride). Fifteen nmol of library was dissolved in 1000 ul of binding buffer (4.5 g/liter glucose, 5 mM MgCl_2_, 0.1 mg/ml tRNA and 1 mg/ml BSA, all in Dulbecco's PBS with magnesium chloride and calcium chloride). The DNA pool was denatured at 95°C for 5 min and quickly cooled on ice for 10 min. The pool was then incubated with the cells at 4°C on rocker for 1 hour. After incubation, the cells were washed three times with washing buffer to remove unbound sequences. Five hundred microliters of binding buffer was added and the cells scraped to recover cell/DNA complexes. The cell-DNA complex was heated at 95°C for 15 min and the mixture centrifuged at 14000 rpm to pellet the cell debris. The supernatant containing the ssDNA was recovered and amplified by PCR using FITC- and biotin-labeled primers to increase the number of copies of individual sequences. A preparative PCR was performed using the amplified pool as the template. The selected sense ssDNA strands were separated from the biotinylated antisense ssDNA by alkaline denaturation and affinity purification with streptavidin-coated Sepharose beads (GE Healthcare Bio-Sciences Corp., Piscataway, NJ, USA). The ssDNA was dried and then resuspended in binding buffer to a final concentration of 1 µM. The pools were denatured at 95°C, snap cooled and used to perform the second round of selection using the same procedure as described for first selection. After washing, the binding sequences were eluted by heating, and the recovered ssDNA was used to perform negative selection using HCT 116. In the control selection, cells were cultured in a 100 mm ×20 mm culture dish. Similarly, cells were washed and incubated with the eluted DNA pool. After incubation, the non-binding sequences in the incubation buffer were recovered. The pool was amplified by PCR using FITC- and biotin-labeled primers, and then PCR product was used to prepare ssDNA.

The entire selection process was repeated until significant enrichment was obtained for the positive cell line (16 rounds) when assayed by flow cytometry. During the selection, the size of the positive cell dish was changed to 60 mm ×15 mm, but that of the control cell line was maintained. Also, the stringency of selection was increased by (i) increasing the volume of washing buffer (ii) increasing washing time and (iii) increasing the amount of FBS from 10% (from 4^th^ round) to 20%. Three enriched pools were amplified by PCR using unlabeled primers and the PCR products cloned into *Escherichia coli* using TOPO TA cloning Kit for sequencing (Invitrogen, Carlsbad, CA, USA). The positive clones were sequenced.

In a parallel approach, different oligonucletide libraries were used to perform another selection with DLD-1, but without negative selection and HCT 116 positive using HT-29 as control. With regards to the HCT 116 selection, our previous study revealed that the cell line binds to Sgc8, a CCRF-CEM aptamer [23], with high fluorescence signal, an indication of high expression of the target PTK7. Therefore, prior to the selection, we introduced 10-fold excess of unlabeled Sgc8 in order to block Sgc8 binding sites on PTK7 and, consequently, avoid selecting for the same target again. The enriched pools were also cloned and sequenced. The rationale of this approach is to generate a panel of aptamers that would have differential binding recognition to colorectal cancers.

### Binding assays

#### 1. Enrichment of selected pool

Flow cytometry was used in all the binding assays to monitor the process and enrichment of the selection pools. Prior to monitoring, DLD-1, HT-29 or HCT 116 cells were cultured overnight. Cells were dissociated either by non-enzymatic cell dissociation solution (MD Biomedicals) or by mild short time (30–60 sec) trypsin treatment at room temperature. Dissociated cells were washed and incubated with 250 nM final concentration of the FITC-labeled ssDNA selection pools at 4°C in 100 µl incubation volume. After washing, the pellets were resuspended in 200 µl washing buffer, and the fluorescence intensity was determined by FACScan cytometer (BD Immunocytometry Systems) by counting 15,000 events. The unselected DNA library labeled with FITC was used as the background signal.

#### 2. Assessment of potential aptamer candidates

The initial assessment of potential aptamer candidates was done with RCA reaction products obtained from sequencing in different 96-well plates. The choice of which wells (sequence) to test was based on the homology of the sequence alignment using ClustalX18.3. Products from representative wells were diluted with 100 µl of H_2_0 before use. Aliquots were amplified by PCR using FITC- and biotin-labeled primers. The PCR products were used to prepare ssDNA, and the sense strand containing the FITC was used for binding assay by flow cytometry. Sequences that showed recognition with positive cells were chemically synthesized and labeled with biotin. Biotin was used to label the synthesized ssDNA so that the binding signal could be detected with any streptavidin-conjugated fluorophore applicable to flow cytometry and microscopy.

#### 3. Screening of potential aptamer candidates and binding affinities

The screening of potential aptamers and the binding affinity of the successful aptamer candidates were done by flow cytometry using biotin-labeled aptamer, and the signal was detected with streptavidin-R-PE conjugate (0.5 mg R-PE at 0.25 mg/mL SAV, Invitrogen) or streptavidin-PE-Cy5.5 (.2 mg/mL). To determine the binding affinity of the aptamers, positive cells were cultured overnight and the cells dissociated using non-enzymatic dissociation buffer and/or short time (30 sec) trypsin treatment. Cells were washed and incubated with varying concentrations (0.10 nM –500 nM final concentration) of biotin-labeled aptamer in a 200 µl volume of binding buffer containing 10% FBS. After 10 min of incubation, cells were washed twice with washing buffer and then incubated with 100 µl PE-streptavidin conjugate or streptavidin-PE-Cy5.5 at a final dilution of 1∶400 dilution (optimized). This was incubated for 10 min and then washed twice with 1200 µl washing buffer. The cell pellets were resuspended in 200 µl washing buffer and analyzed by flow cytometry. The biotin-labeled unselected library was used as a negative control to determine the background binding. All binding assays were done in duplicate. The mean fluorescence intensity of the unselected library was subtracted from that of the corresponding aptamer with the target cells to determine the specific binding of the labeled aptamer. The apparent equilibrium dissociation constant (Kd) of the aptamer-cell interaction was then obtained by fitting the dependence of intensity of specific binding on the concentration of the aptamers to the equation Y  =  B max X/(kd + X), using Sigma Plot (Jandel, San Rafael, CA).

### Confocal microscopy

The binding of the selected aptamers with the cells was further assessed by confocal microscopy. The binding assay was similar to the selection procedure. Here, DLD-1 or HCT 116 cells were seeded in a 35 mm petri dish, 10 mm microwell (MatTek Corporation), and cultured overnight. The cells showing more than 60% confluence were carefully washed and then incubated with the aptamers or control library at a final concentration of 250 nM. After incubation, cells were carefully washed before incubation with 1∶200 dilution (optimized) of streptavidin-conjugated Alexa Fluor 633 (Invitrogen) or 1∶400 dilution of PE-streptavidin conjugate for 10 min. Excess probes were washed off and the signal detected with confocal microscopy (FV500-IX81 confocal microscope, Olympus America Inc., Melville, NY), with 40× oil immersion objective (NA = 1.40, Olympus, Melville, NY). Excitation wavelength and emission filters were as follows: PE, 488 nm laser line excitation, emission BP520; and Alexa Fluor 633 nm laser line excitation, emission LP650 filter.

### Selectivity

The recognition of all the selected aptamers was tested on the three colorectal cancer cell lines (DLD-1, HCT 116 and HT-29), normal colon cell lines (FHC, CC 18Co) as well as HBE135 E6/E7 (normal epithelial cell line). Although these aptamers were developed using colorectal cancer cell lines, the possibility of selecting for targets that are also present on other cancers could not be ruled out. Therefore, the selectivity was further extended to other cell lines from different cancers, including K562 (CML), NB4 (APL), HL60, KG-1 (AML), Ramos (Burkitt's lymphoma), CCRF-CEM (ALL), Hela (cervical) and NCI-H23, H1979, H661 (small cell lung cancer), Ludlu (squamous cells), CAOV3, (ovarian cancer cell line) and U87MG (brain tumor). These cell lines were used in binding assays as described above. In all the assays, the final concentration of the each aptamer was 250 nM.

### Competition assays

These selections generated a number of aptamers with varying binding patterns and florescence signal intensities to the target cell DLD-1. Competition assays were done to assess if any of these aptamers would bind to the same target. In general, cell-SELEX can identify multiple aptamers for multiple targets as well as multiple aptamers for a single target in a single selection. These competition assays were designed based on the aptamers' selectivity results. One set was designed for the aptamers that showed selective binding to only the target DLD-1 cells, whereas the other one was designed for all the other aptamers. In each case, the unlabeled aptamer competitor was incubated with DLD-1 cells in 10-fold excess final concentration (2.5 µM). After incubation, the other FITC-labeled aptamer was added at a final concentration of 250 nM. As positive control to assess the effectiveness of the assay, the same aptamer, with both labeled and unlabeled, was used. The cells were washed and fluorescence intensity determined by flow cytometry, as described.

### Effect of temperature and culture medium on the binding of aptamers

The ability of aptamers to recognize target at physiological temperature is important, especially where these aptamers were generated at lower temperatures. We therefore performed binding assays at 37°C to verify the stability of the binding of the aptamer to its target. Before the binding assays, all reagents and buffers were maintained at room temperature. Again, all centrifugations were done at room temperature, but the actual binding was done at 37°C. Briefly, DLD-1 cells cultured for 24 hours were dissociated with short time trypsin treatment. Cells were washed and incubated with biotin-labeled aptamer at the final concentration of 250 nM. After incubation, the cells were washed and incubated with streptavidin-PE or streptavidin PE-Cy5.5 for 10 mins. The cells were washed and the fluorescence intensity determined.

The initial selection, monitoring and all the reported assays were done in phosphate buffer. We therefore further assessed if the aptamers could maintain the binding structure in culture medium to recognize the target. Here binding assays were performed in culture medium, and incubation was done both at 4°C and 37°C.

### Normal human colon specimen

We tested the recognition pattern of these aptamers on fresh normal human colon specimen. The fresh tissue was chopped into small pieces and washed with PBS. The pieces were then incubated in dissociation solution for 10 min. After a substantial number of cells were released into solution, the supernatant was collected and the cells pelleted. This was used in the binding assay and detected by flow cytometry as described.

## Supporting Information

Figure S1 Binding assays showing the interaction of DNA selected pools with DLD-1 cells dissociated using non enzymatic dissociation solution (A) and short time trypsin (B). Black histogram (unselected library background), red (13th selected pool) and blue (14th selected pool).
(1.27 MB TIF)

Figure S2 Flow cytometry dotplots showing the interaction of the RCA products with DLD-1 cells. A threshold based on fluorescence intensity of FITC in the flow cytometry was set so that about 5% of cells incubated with the FITC-labeled DNA library represent fluorescence intensity background (lower right quadrant), and the binding event was assessed based on the percentage of cells binding over the threshold.
(1.41 MB TIF)

Figure S3 Flow cytometry dot plot showing the interaction of aptamer with normal human colon cells.
(1.83 MB TIF)

Figure S4 Assessment of the effect of binding KDED19, KC2D4, and KC2D8 to DLD-1 cells in the presence of excess of unlabeled Sgc8. Red histogram (control background); green (aptamer binding without excess of unlabeled Sgc8) and blue (aptamer binding in the presence of excess unlabeled Sgc8).
(1.35 MB TIF)

Figure S5 Assessment of the binding of selected aptamers at 37°C. Red color represents cell background; green (unselected library) and blue (aptamer signal).
(1.39 MB TIF)

Figure S6 Selected aptamers were incubated with cells using culture medium RPMI-1640 as the binding medium. Red (unselected library background); Blue (aptamer signal using PBS binding buffer) and Green (aptamer signal in RPMI-1640).
(1.38 MB TIF)

Text S1 (0.04 MB DOC)
