# Supplementary figures and images for: DNA Aptamers as Molecular Probes for Colorectal Cancer Study
Source: PLoS One. 2010 Dec 10;5(12):e14269. doi: 10.1371/journal.pone.0014269 (PMC3000811; doi:10.1371/journal.pone.0014269)

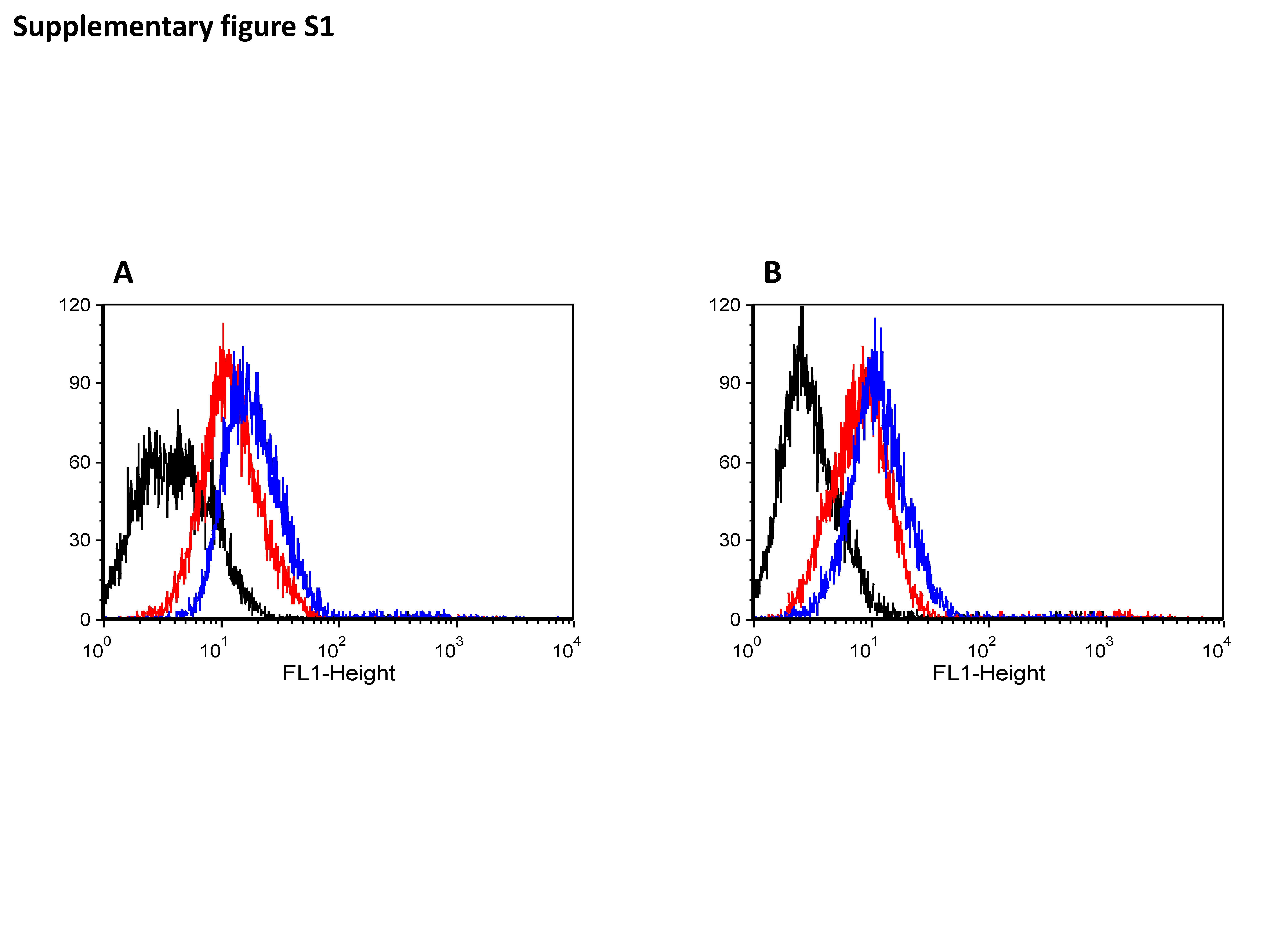

Supplement: Figure S1 — Binding assays showing the interaction of DNA selected pools with DLD-1 cells dissociated using non enzymatic dissociation solution (A) and short time trypsin (B). Black histogram (unselected library background), red (13th selected pool) and blue (14th selected pool). (1.27 MB TIF) [file pone.0014269.s001.tif]

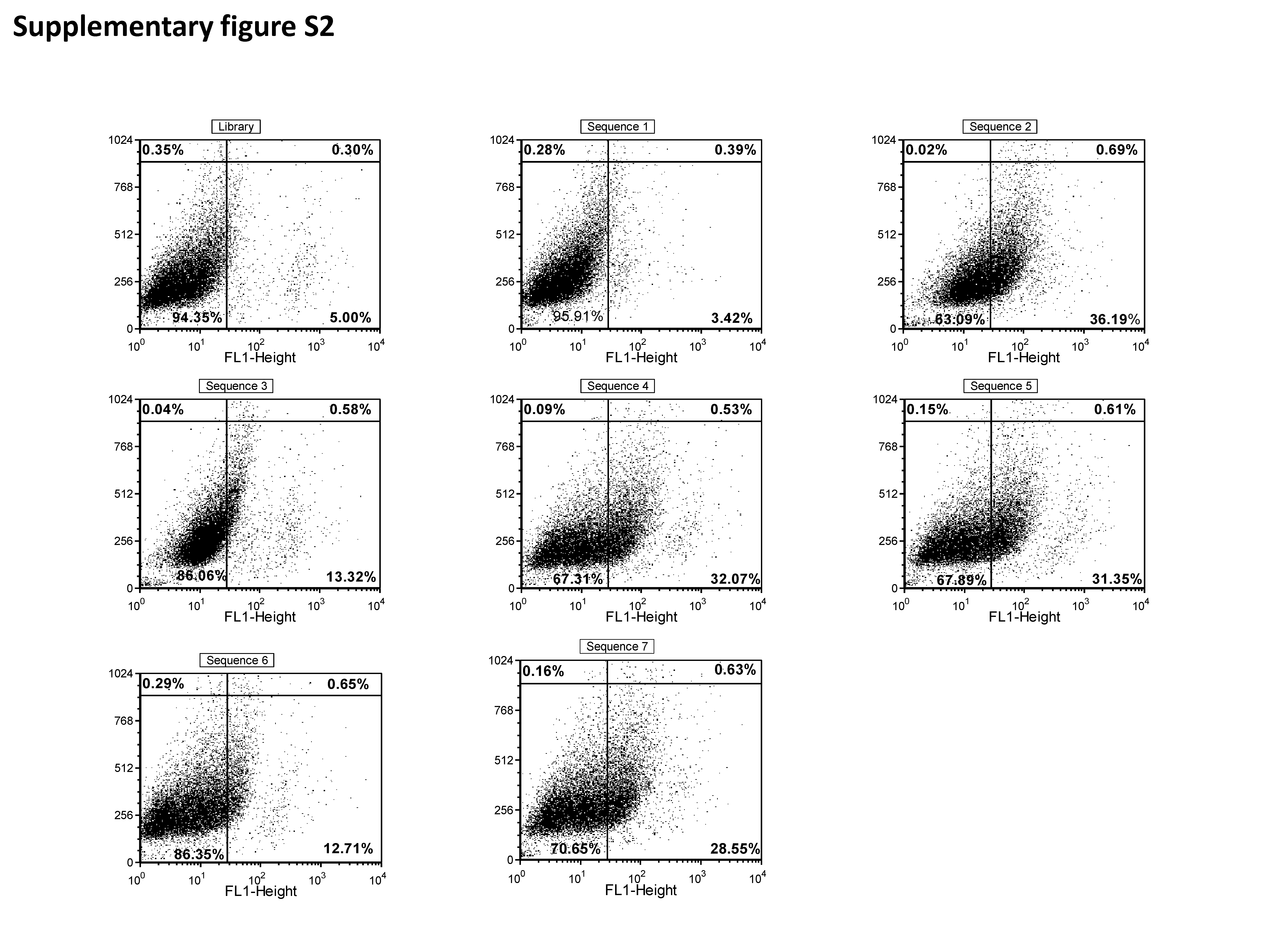

Supplement: Figure S2 — Flow cytometry dotplots showing the interaction of the RCA products with DLD-1 cells. A threshold based on fluorescence intensity of FITC in the flow cytometry was set so that about 5% of cells incubated with the FITC-labeled DNA library represent fluorescence intensity background (lower right quadrant), and the binding event was assessed based on the percentage of cells binding over the threshold. (1.41 MB TIF) [file pone.0014269.s002.tif]

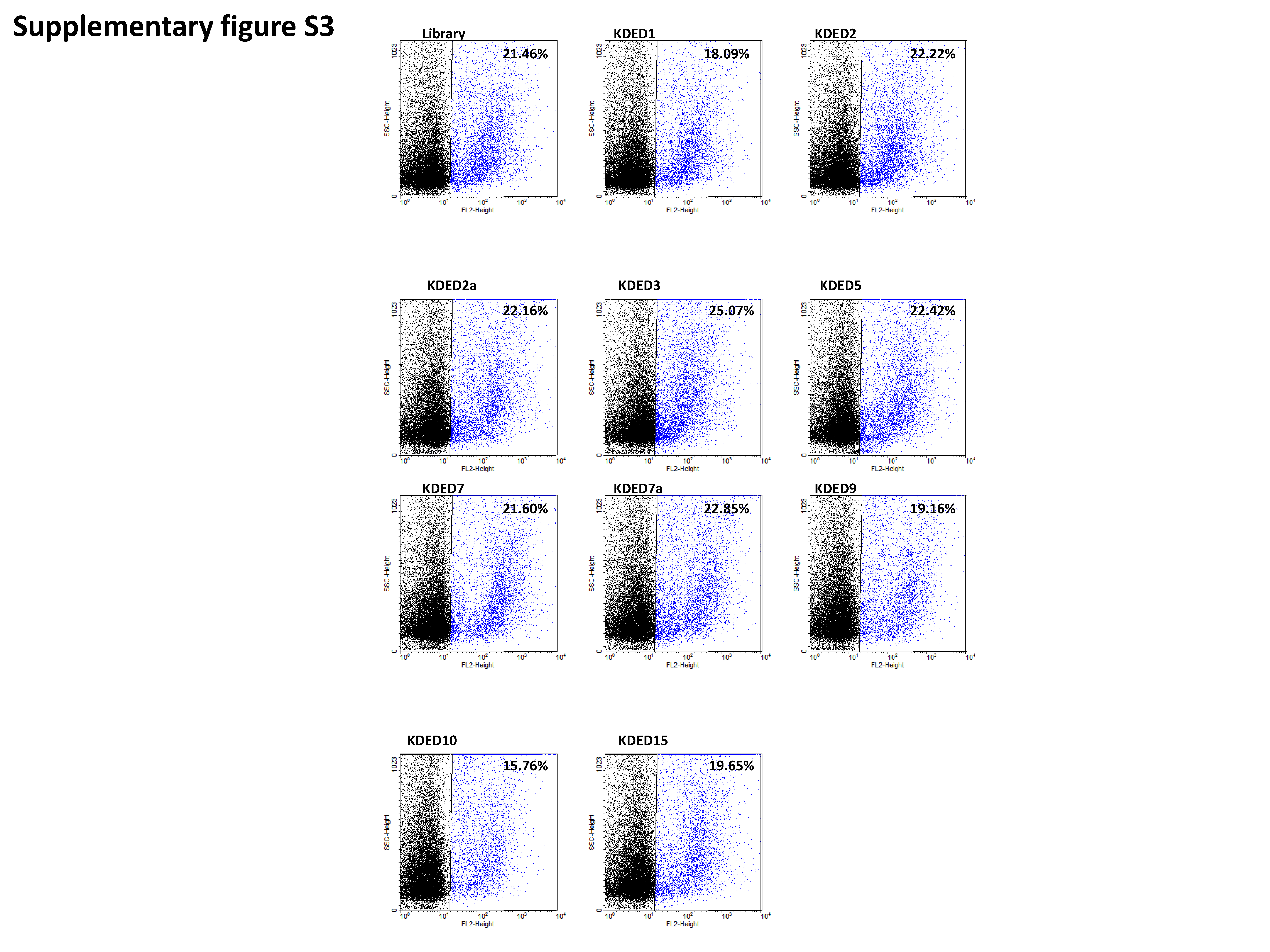

Supplement: Figure S3 — Flow cytometry dot plot showing the interaction of aptamer with normal human colon cells. (1.83 MB TIF) [file pone.0014269.s003.tif]

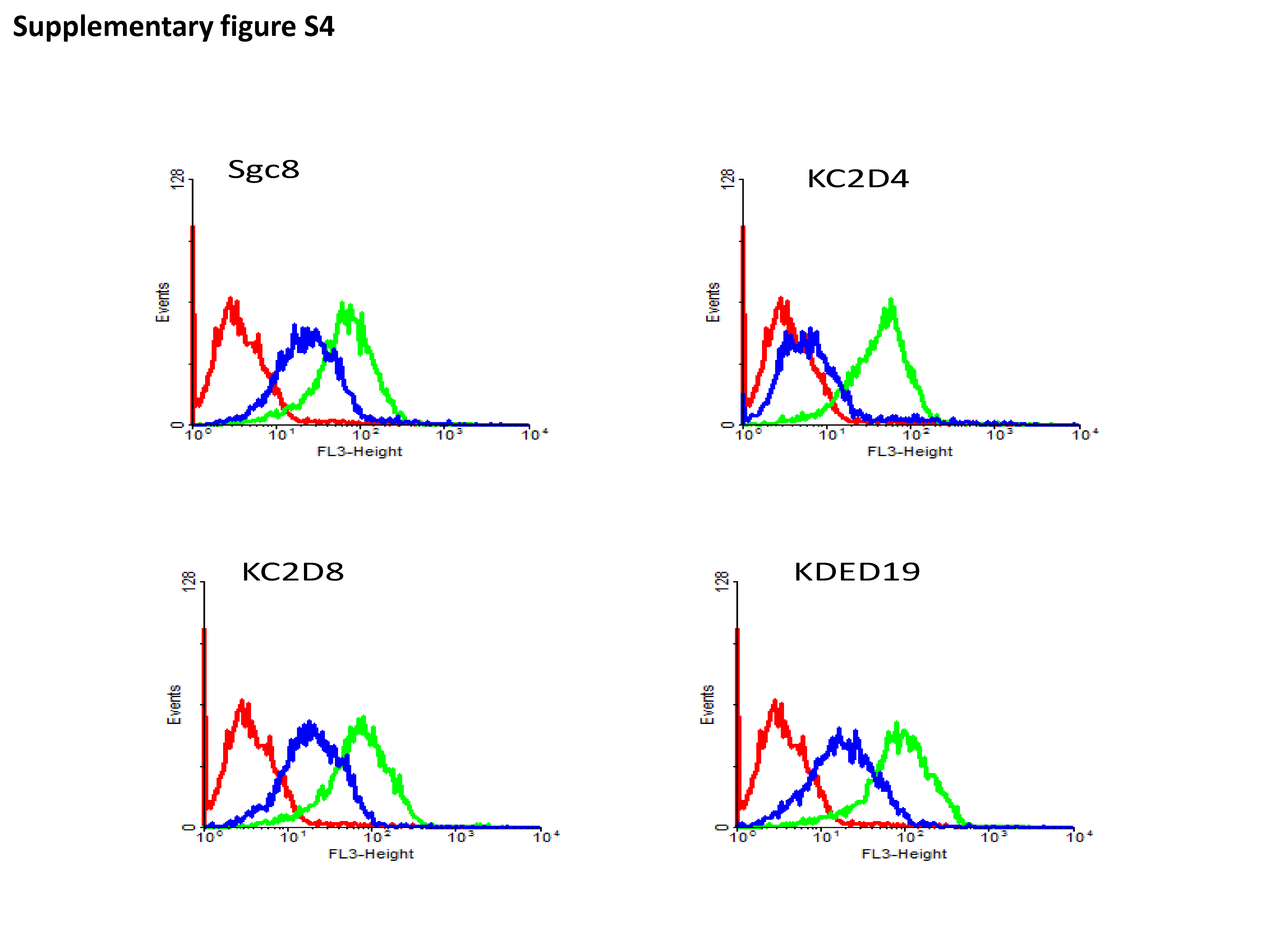

Supplement: Figure S4 — Assessment of the effect of binding KDED19, KC2D4, and KC2D8 to DLD-1 cells in the presence of excess of unlabeled Sgc8. Red histogram (control background); green (aptamer binding without excess of unlabeled Sgc8) and blue (aptamer binding in the presence of excess unlabeled Sgc8). (1.35 MB TIF) [file pone.0014269.s004.tif]

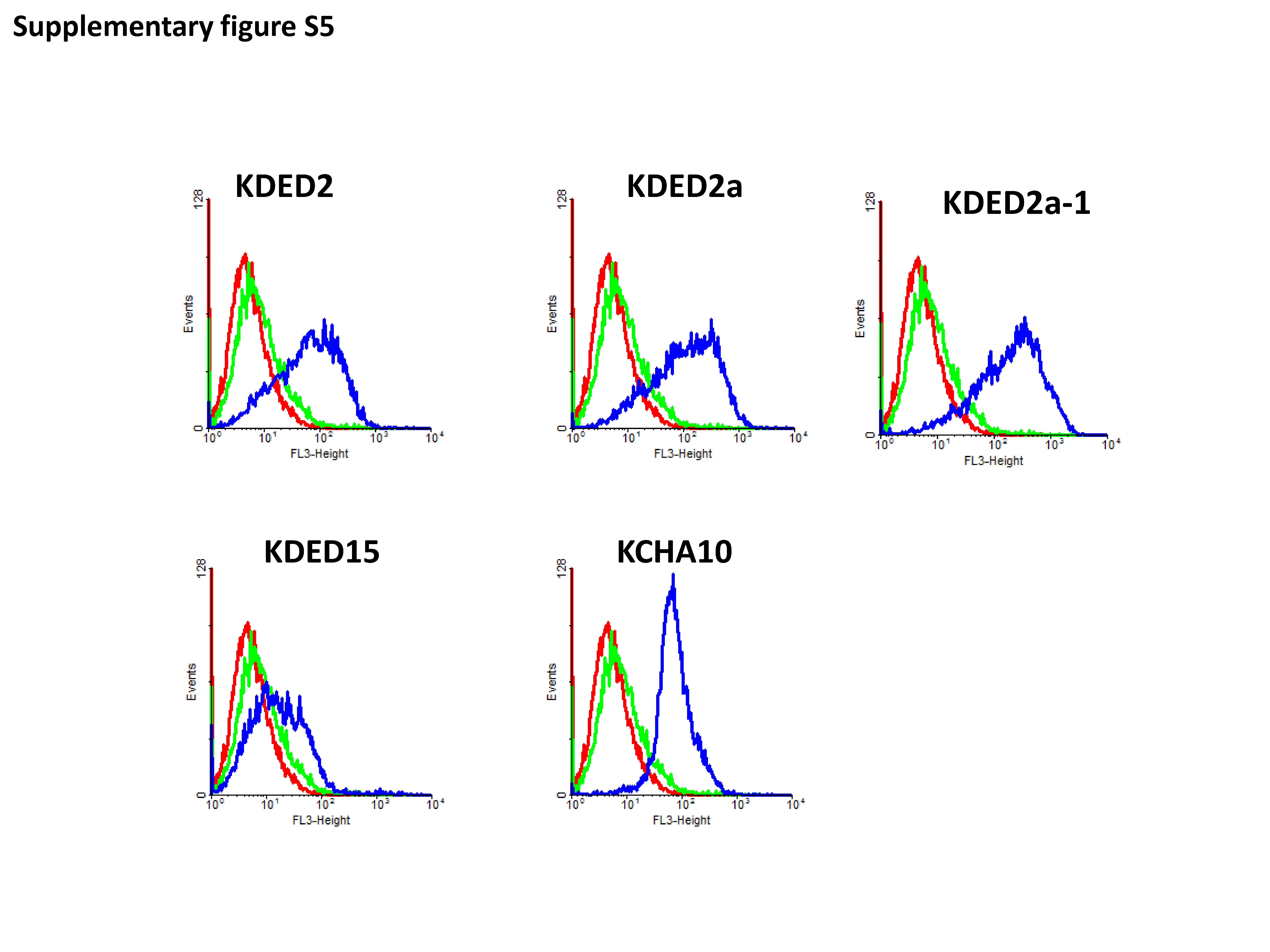

Supplement: Figure S5 — Assessment of the binding of selected aptamers at 37°C. Red color represents cell background; green (unselected library) and blue (aptamer signal). (1.39 MB TIF) [file pone.0014269.s005.tif]

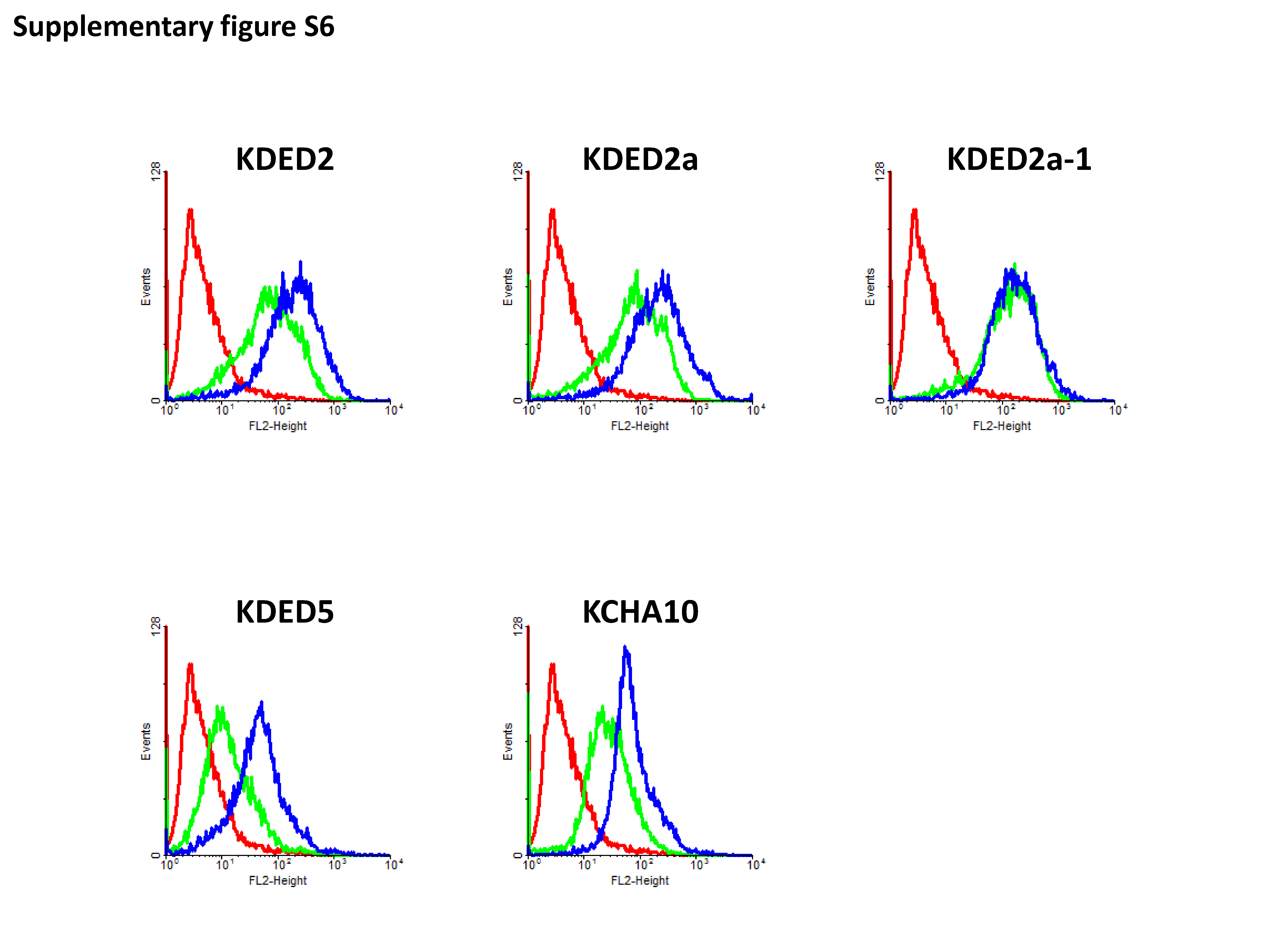

Supplement: Figure S6 — Selected aptamers were incubated with cells using culture medium RPMI-1640 as the binding medium. Red (unselected library background); Blue (aptamer signal using PBS binding buffer) and Green (aptamer signal in RPMI-1640). (1.38 MB TIF) [file pone.0014269.s006.tif]
