# Supplementary material for: DNA Aptamers as Molecular Probes for Colorectal Cancer Study
Source: PLoS One. 2010 Dec 10;5(12):e14269. doi: 10.1371/journal.pone.0014269 (PMC3000811; doi:10.1371/journal.pone.0014269)
Supplement: Text S1 — (0.04 MB DOC) [file pone.0014269.s007.doc]

**Title: DNA aptamers as molecular probes for colorectal cancer study**

**Kwame Sefah, Ling Meng, Dalia Colon, Elizabeth Jimenez, Chen Liu and Weihong Tan**

**Supplementary information**

**Dissociation of adherent cells**

Flow cytometry is one of the easiest and simplest means of assessing the interaction of dye-labeled probes with the surface markers of live cells. It is even more convenient if the cells for the analysis are suspension. However when the cells are adherent monolayer, they need to be dissociated from the culture flask/dish before they can be used. Even with the introduction of this extra step, flow cytometry has relatively short turn-around time and high sensitivity. Therefore during process flow cytometry was used to assess the interaction of the pools with the cells. The method of dissociation is very important since this can influence the binding studies. Traditionally trypsin solution has been widely used for cell dissociation during culture, but the procedure can be damaging to membrane proteins especially in the event of prolong exposure. To maintain the integrity of the membrane surface proteins during cell dissociation, various commercial non enzymatic buffers have been formulated but most of these are very slow and often times damaging to the cells. In view of this we optimized the dissociation procedures of the adherent cells using both trypsin and non enzymatic dissociation buffers and the results are depicted in figure S1.

Cells were split and seeded at very low density and cultured for 24 hours. Prior to the assay, the culture medium was removed from the culture dish and cells were removed with non enzymatic dissociation buffer or short-term (30sec-1min) trypsin treatment. Cells were harvested and then used for the binding studies. The histograms presented here show that short time trypsin treatment did not affect the binding of the selected pools. This procedure has been outlined in Sefah et al., 2010 [1].

**Testing of RCA products to identify potential aptamer candidates**

The use of rolling cycle amplification products generated in the sequencing procedure is one of the fastest means of identifying potential aptamer candidates. After sequencing and alignment of the randomized regions, different families were obtained. Members from each of these families were tested using their RCA products. Here, the sample was collected from the 96-well plate and used as template to prepare PCR using FITC- and Biotin-labeled primers. The FITC-sense strands were separated from the biotinylated antisense ssDNA by affinity purification with streptavidin-coated Sepharose beads (GE Healthcare Bio-Sciences Corp., Piscataway, NJ, USA) and alkaline denaturation.. The ssDNA was dried and then resuspended in binding buffer to a final concentration of 500nM. The products were used for binding studies using DLD-1 cells, using ssDNA library as control, all at a final concentration of 250nM. Supplementary figure S2 shows the interaction of the some of the ssDNA with the target cell line. From the dotplots in supplementary figure S2 sequences 2, 4, 5 and 7 showed more cells binding over the set threshold with percentage in the lower right 36.16, 32.07, 31.35 and 28.55 respectively. With these obvious signals, the sequences were chemically synthesized. Sequences 1 did not show any obvious signal whilst sequence 6, though did not give signal above the threshold, showed complete shift of all the cells to the right, an indication of recognition. As a result sequence 6 was also chemically synthesized. In the end the sequences 2, 4, 5, 6 and 7 represented the aptamers KDED2, KDED7, KDED5, KDED10 and KDED18 respectively. The others aptamers were identified using similar approach.

**Aptamer interaction with normal colon clinical specimen**

The ability of a probe to selectively differentiate tumor cells from the adjoining normal cells is important for tumor detection as well as targeted therapy. It is therefore necessary to determine if the individual aptamers generated by cell-SELEX, which commonly bind to unknown targets, also bind to normal cells, especially if the normal cell was not used for negative selection. Figure S3 shows the data obtained with aptamers generated by cell-SELEX using DLD-1 as the target cell line.

Fresh human colon tissue was obtained from the Pathology Department of the Shands hospital at the University of Florida. The tissue was minced and the cells dissociated with dissociation buffer. The suspension was passed through cell strainer (40µm) and the cell suspension used in flow cytometry binding assay with the indicated aptamers. The fluorescence background was set with unselected library. A relatively high fluorescence background was observed for the control (20% of total cell number). This might be due the presence of dead cell as tissue dissociation processes commonly generate some dead cells. However as shown the fluorescence signals of the aptamers were similar to that of the library. These suggest that none of the aptamers tested could recognize surface markers of the normal colon cells. This observation is important since it will allow the opportunity to use these aptamers for clinical colorectal cancer specimen, which is usually not 100% pure diseased cells but contains some percentage of normal tissues as will. Thus, irrespective of the percentage that is normal cells, the aptamer binding will not be influenced.

**Competition study between generated aptamer and previously evolved aptamer**

The observation that some of the generated aptamers can bind to CCRF-CEM and not Ramos (Burkitt’s lymphoma) aroused the interest of determine if these aptamers can compete with an already generated aptamer to CCRF-CEM [2], which used Ramos as the control. The figure below demonstrates the observation. Sgc8 is an aptamer evolved for CCRF-CEM and the target of this aptamer was later identified to be PTK7 [3], a non receptor tyrosine kinase. Incidentally, PTK 7 had earlier on been described in colon cells. Based on this information, prior to the development of aptamers for the colorectal cancer cell lines, Sgc8 was tested against HCT 116, DLD-1 and HT-29. Sgc8 bound to HCT 116 with very high signal intensity but close to background signal was observed for DLD-1 and HT-29. Consequently during selection unlabeled Sgc8 was used for blocking in only the HCT 116 and not the DLD-1. After developing the aptamers, they were tested against wide variety of tumor cell lines and it was observed that three of the aptamers generated for DLD-1 also bound to CCRF-CEM but not to Ramos, a negative control cell line for Sgc8. We therefore did a competition assay to determine if these Sgc8 could compete with these aptamers. In the assay, unlabeled Sgc8 was first incubated with the DLD-1 cells at a concentration of 2.5μM, followed by the biotin-labeled aptamer at a final concentration of 250nM. After incubation and washing, the binding signal was detected with stretavidin-PE-cy5.5. As shown in the figure the binding of all the three aptamers was affected by the presence of excess Sgc8. This data suggest preliminary that these aptamers and Sgc8 might be binding to the same target.

**Aptamers binding at different assay conditions**

The development of aptamers that will bind to the target at varying conditions especially at physiological temperature and in culture medium is important. This will increase the flexibility with which these aptamers can be adopted and implemented in many assay platforms. We therefore assessed the binding of the aptamers at 37^o^C, in culture medium and under both conditions simultaneously. As shown in supplementary figure S5, KDED2, KDED2a, KDED2a-1 (same aptamer but different sequence lengths) and KCHA10 maintained significant binding to DLD-1 cells. The signal strengths were not significantly different from the assays at 4^o^C. On the other hand the other aptamers had reduced signal intensity to DLD-1. The signal difference might be due to the differential affinity of individual aptamers. For instance KDED2 (better affinity) and KDED15 belong to the same family but KDED15 did not show significant binding at 37^o^C. In the RPMI-1640 experiments, KEDE2, KDED2a and KDED2a-1 showed equal binding strength even when the incubations were done at 37^o^C. KDED5 and KCHA10 showed reduced but significant binding than the rest of the aptamers (supplementary figure S6).

Before incubation with the cells, 100μl of aptamers (500nM) in binding buffer were placed in incubator at 37^o^C for 10 min. 100 μl of cell suspension was then added to give a final concentration of 250nM. Binding mixture was incubated for 10min at 37^o^C. Cells were washed and the binding was assessed by fluorescence signal in flow cytometry.

**References**

1. Sefah K, Shangguan D, Xiong X, O’Donoghue MB, Tan W (2010) Development of DNA aptamers using Cell-SELEX. Nature Protocols 5: 1169-1185.

2. Shangguan D, Li Y, Tang Z, Cao ZC, Chen HW, et al. (2006) Aptamers evolved from live cells as effective molecular probes for cancer study. Proc Natl Acad Sci (U S A) 103: 11838–11843.

3. Shangguan D, Cao Z, Meng L, Mallikaratchy P, Sefah K, et al. (2008) Cell-Specific Aptamer Probes for Membrane Protein Elucidation in Cancer Cells. J Proteome Res 7: 2133-2139.

**Supplementary figure S1:** Binding assays showing the interaction of DNA selected pools with DLD-1 cells dissociated using non enzymatic dissociation solution (A) and short time trypsin (B). Black histogram (unselected library background), red (13^th^ selected pool) and blue (14^th^ selected pool).

**Supplementary figure S2:** Flow cytometry dotplots showing the interaction of the RCA products with DLD-1 cells. A threshold based on fluorescence intensity of FITC in the flow cytometry was set so that about 5% of cells incubated with the FITC-labeled DNA library represent fluorescence intensity background (lower right quadrant), and the binding event was assessed based on the percentage of cells binding over the threshold.

**Supplementary figure S3:** Flow cytometry dot plot showing the interaction of aptamer with normal human colon cells.

**Supplementary figure S4:** Assessment of the effect of binding KDED19, KC2D4, and KC2D8 to DLD-1 cells in the presence of excess of unlabeled Sgc8. Red histogram (control background); green (aptamer binding without excess of unlabeled Sgc8) and blue (aptamer binding in the presence of excess unlabeled Sgc8).

**Supplementary Figure S5**: Assessment of the binding of selected aptamers at 37^o^C. Red color represents cell background; green (unselected library) and blue (aptamer signal).

**Supplementary figure S6:** Selected aptamers were incubated with cells using culture medium RPMI-1640 as the binding medium. Red (unselected library background); Blue (aptamer signal using PBS binding buffer) and Green (aptamer signal in RPMI-1640).
